# Large-Scale Expansion of Human Mesenchymal Stem Cells

**DOI:** 10.1155/2020/9529465

**Published:** 2020-07-15

**Authors:** Muhammad Najib Fathi Bin Hassan, Muhammad Dain Yazid, Mohd Heikal Mohd Yunus, Shiplu Roy Chowdhury, Yogeswaran Lokanathan, Ruszymah Bt Hj Idrus, Angela Min Hwei Ng, Jia Xian Law

**Affiliations:** ^1^Tissue Engineering Centre, Faculty of Medicine, Universiti Kebangsaan Malaysia Medical Centre, Jalan Yaacob Latif, 56000 Kuala Lumpur, Malaysia; ^2^Department of Physiology, Faculty of Medicine, Universiti Kebangsaan Malaysia Medical Centre, 56000 Kuala Lumpur, Malaysia

## Abstract

Mesenchymal stem cells (MSCs) are multipotent stem cells with strong immunosuppressive property that renders them an attractive source of cells for cell therapy. MSCs have been studied in multiple clinical trials to treat liver diseases, peripheral nerve damage, graft-versus-host disease, autoimmune diseases, diabetes mellitus, and cardiovascular damage. Millions to hundred millions of MSCs are required per patient depending on the disease, route of administration, frequency of administration, and patient body weight. Multiple large-scale cell expansion strategies have been described in the literature to fetch the cell quantity required for the therapy. In this review, bioprocessing strategies for large-scale expansion of MSCs were systematically reviewed and discussed. The literature search in Medline and Scopus databases identified 26 articles that met the inclusion criteria and were included in this review. These articles described the large-scale expansion of 7 different sources of MSCs using 4 different bioprocessing strategies, i.e., bioreactor, spinner flask, roller bottle, and multilayered flask. The bioreactor, spinner flask, and multilayered flask were more commonly used to upscale the MSCs compared to the roller bottle. Generally, a higher expansion ratio was achieved with the bioreactor and multilayered flask. Importantly, regardless of the bioprocessing strategies, the expanded MSCs were able to maintain its phenotype and potency. In summary, the bioreactor, spinner flask, roller bottle, and multilayered flask can be used for large-scale expansion of MSCs without compromising the cell quality.

## 1. Introduction

Mesenchymal stem cells (MSCs) are multipotent stem cells which can be isolated from various tissue sources such as bone marrow [1], adipose tissue [2], and umbilical cord [3]. MCSs are able to self-renew and can be induced to differentiate into adipocytes, chondrocytes, osteocytes, hepatocytes, tenocytes, and cardiomyocytes [2, 4, 5]. MSCs can modulate the immunoreactivity through mechanisms such as suppression of T-cells and lymphocyte proliferation [6, 7]. In addition, MSCs also possess antioxidative, antiapoptotic, antifibrosis, and proangiogenesis properties [8]. Thus, MSCs have remarkable clinical potential especially in immune modulation and tissue regeneration. In fact, MSCs have been evaluated in many clinical trials for the treatment of immune-mediated diseases and tissue injuries. Diseases that have been treated with MSCs include liver diseases, peripheral nerve damage, graft-versus-host-disease, autoimmune diseases, diabetes mellitus, and heart diseases [9, 10].

A crucial limitation in therapeutic application of MSCs is the low amount of MSCs in all tissues and the quantity of isolated MSCs being insufficient for clinical use. A dosage of 2 × 10^6^ cells/kg body weight is commonly given to the patients [6, 7]. For certain patients and diseases, multiple administrations of MSCs up to several hundred million cells are needed to achieve the desired therapeutic effect [11, 12].

MSCs can be expanded *in vitro* using a cell culture plate and flask to obtain the sufficient cell number needed for experimental purposes. However, a similar strategy is not ideal for expansion of MSCs meant for clinical use as the cell number needed is much higher. More manpower and incubator space are needed when performing large-scale cell expansion using a cell culture flask. Apart from being ineffective, large-scale expansion using a cell culture flask also affects the cell quality as MSCs expanded *in vitro* for a long period of time may lose their stem cell characteristics [13]. Previous studies also reported that MSC proliferation and differentiation potential decreased when they reached a higher passage number [14]. Thus, identification of an effective large-scale expansion technique is very important to obtain the huge number of cells in a short period of time and in a cost-effective manner without compromising the cell quality.

In this review, we identified the articles reporting the large-scale expansion of MSCs via systematic literature search. A total of 4 bioprocessing strategies, i.e., bioreactor, spinner flask, roller bottle, and multilayered flask, were found to be used for large-scale expansion of MSCs, and all data reported in these articles were extracted, analyzed, and discussed.

## 2. Methods

### 2.1. Search Strategy

A systematic literature search was carried out to identify suitable articles reporting large*-*scale expansion of human MSCs *in vitro*. Literature search was performed using keywords, (1) human AND (2) mesenchymal stem cells OR mesenchymal stromal cells OR MSCs AND (3) large-scale OR scale-up, in a sentence of ((*human*) *AND* ((*mesenchymal AND stem AND cells*) *OR* (*mesenchymal AND stromal AND cells*) *OR* (*MSCs*)) *AND* ((*large AND scale AND expansion*) *OR* (*up AND scaling*))) in the Medline and Scopus databases. Next, only the literature articles reported in English language were selected. The articles must also meet the inclusion and exclusion criteria to be included in this study.

The first inclusion criterion is that the articles are working on human MSCs. Secondly, the articles described the large-scale expansion of human MSCs. Thirdly, the articles provide detailed information on the expansion process, including the source of MSCs, cell seeding density, expansion method, medium composition, culture period, and total cell yield. Lastly, the articles characterized the expanded cells in accordance with the minimal criteria established by the International Society for Cellular Therapy (ISCT). Review articles and proceedings were excluded. In addition, articles describing the large-scale expansion of MSCs using the standard culture flask, i.e., T-25, T-75, and T-175 flasks, were also excluded.

### 2.2. Data Extraction

Data were extracted from selected articles by two authors independently. The articles were selected through 3 layers of screening, i.e., title screening, abstract screening, and whole article screening, to exclude articles that did not fulfill the inclusion and exclusion criteria. Data were extracted from articles that provide detailed description of at least one large-scale expansion process. For articles reporting multiple large-scale expansion processes, information of all the described expansion processes was collected.

### 2.3. Calculation

Efficiency of large-scale expansion was compared by calculating the expansion fold using the following formula:

(1) $$\begin{array}{c} \text{Expansion ratio}=\frac{\text{Total cell yield}}{\text{Total cell seeded}}. \end{array}$$

Some articles reported the number of cells seeded as the total cell number while others as cell seeding density. For standardization, all data were converted to the total cell number. This is to give an idea on the number of cells needed prior to large-scale expansion as well as to show the total cell yield upon expansion using the specific bioprocessing methods. The total cell number was calculated using the following formula:

(2) $$\begin{array}{c} \text{Total cell number }\left(t\right)=\rho \times A, \end{array}$$

where *ρ* represents cell seeding density or cell yield density and *A* represents the surface area or working volume of the vessel used for cell expansion.

## 3. Results

### 3.1. Literature Search

**Figure 1:**
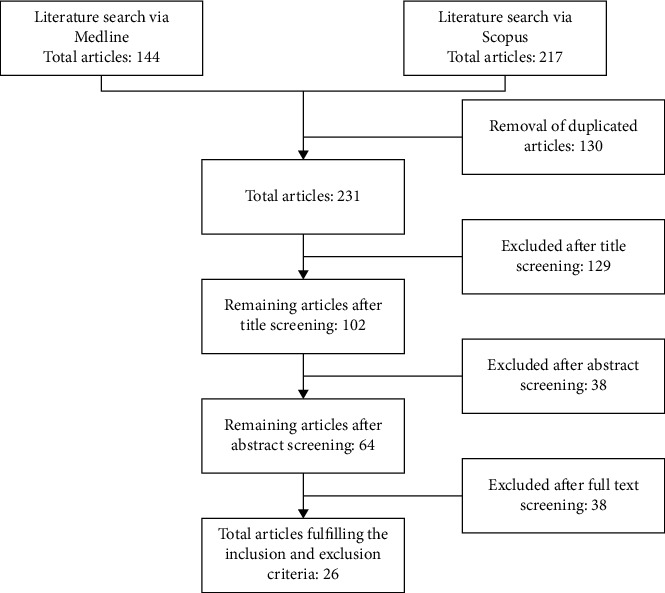
Flow chart of article selection process.

The literature search identified 361 articles: 144 articles were obtained from the Medline database and 217 articles were obtained from the Scopus database. A total of 130 duplicated articles were removed before screening using the inclusion and exclusion criteria. A total of 129 articles were rejected after the title screening because they were not related to large-scale bioprocessing of human MSCs. For the remaining 102 articles screened for the abstract, only 64 articles were selected for thorough full-text screening. Finally, a total of 26 articles were selected for data extraction (Figure 1).

### 3.2. Data Extraction

**Figure 2:**
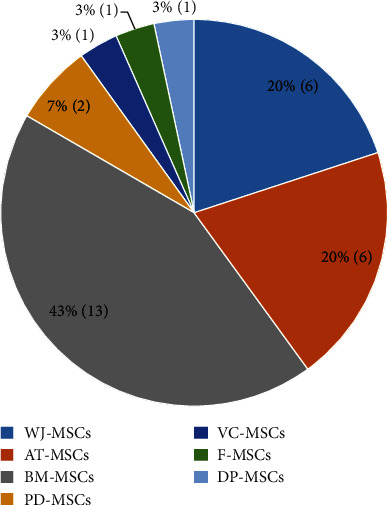
Frequency of the mesenchymal stem cell (MSC) sources in the selected articles. Most of the studies expanded the MSCs derived from bone marrow, adipose tissue, and Wharton's jelly. BM: bone marrow; AT: adipose tissue; WJ: Wharton's jelly; PD: periosteum; VC: villous chorion; F: fetal; DP: dental pulp.

**Figure 3:**
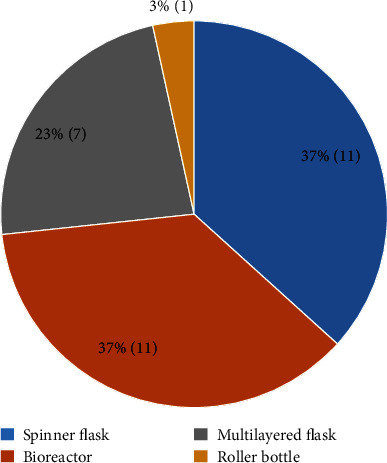
Frequency of the bioprocessing strategies used in the selected studies. Most of the studies used bioreactor, spinner flask, and multilayered flask for large-scale expansion of MSCs.

Data from 26 articles published between 2007 and 2019 were extracted and are summarized in Table 1. The articles described the large-scale expansion of MSCs isolated from 7 different tissue sources, i.e., adipose tissue-derived MSCs (AT-MSCs), umbilical cord matrix- or Wharton's jelly-derived MSCs (WJ-MSCs), bone marrow-derived MSCs (BM-MSCs), periosteum-derived MSCs (PD-MSCs), villous chorion-derived MSCs (VC-MSCs), dental pulp-derived MSCs (DP-MSCs), and fetal MSCs (F-MSCs) (Figure 2). A total of 4 bioprocessing strategies have been used, i.e. bioreactor, spinner flask, roller bottle, and multilayered flask (Figure 3). Four articles described the large scale of expansion of MSCs from multiple sources, and 5 articles used more than 1 bioprocessing method. Most of the articles described the large-scale expansion of MSCs from BM (13 articles, 43%), AT (6 articles, 20%), and WJ (6 articles, 20%), with PD-MSCs appearing in 2 articles (7%) and VC-MSCs, DP-MSCs, and F-MSCs appearing in 1 article (3%) each. Large-scale expansion using the bioreactor, spinner flask, multilayered flask, and roller bottle was described in 11 (37%), 11 (37%), 7 (23%), and 1 (3%) articles, respectively.

### 3.3. Culture Medium Selection

A total of 13 studies reported the use of fetal bovine serum (FBS) at 10 or 15% (*v*/*v*) concentration for large-scale expansion of MSCs of which three studies further supplemented the culture medium with basic fibroblast growth factor (bFGF) ranging from 2 to 10 ng/ml [6, 15, 16]. Another three studies compared the large-scale expansion of MSCs using FBS versus human platelet lysate (HPL) and defined medium [17–19]. All three studies reported that FBS was inferior compared to HPL and defined medium in promoting MSC proliferation. Instead of FBS, seven studies used 5%, 8%, or 10% (*v*/*v*) HPL [17, 19–24], one study used 5% (*v*/*v*) Ultragrow™ [25], and another one used 15% (*v*/*v*) AB human serum as supplement [26]. Defined culture mediums, i.e., MesenCult™-XF medium [27–29], StemPro® MSC SFM XenoFree medium [30–33], Corning® stemgro® hMSC medium [29], and PRIME-XV™ SFM medium [18], were used in seven studies (Table 2). Even though different mediums and medium supplements were used, nonetheless, all the studies reported that the expanded cells maintained its phenotype and trilineage differentiation potential. Five studies showed that the expanded MSCs retained its immunomodulatory properties [6, 7, 20, 34, 35].

### 3.4. Large-Scale Expansion Using Multilayered Flask

**Figure 4:**
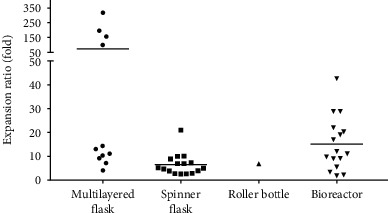
Expansion ratios achieved using different bioprocessing strategies. Multilayered flask and bioreactor can achieve higher expansion ratio compared to spinner flask and roller bottle.

A multilayered flask is a specially designed culture flask that consists of multiple layers of a cell culture-treated surface to provide a large surface area for cell growth. The usage of a multilayered flask saves a lot of incubator space compared to T-75 or T-175 flasks as it is more compact. A few types of multilayered flasks, including Hyperflask, CellSTACK (2-chamber and 5-chamber), and Cell Factory (4-chamber), have been tested for the large-scale expansion of MSCs. The surface area of a multilayered flask varies with types. The hyperflask surface area is 1720 cm^2^, CellSTACK has a surface area ranging from 1272 cm^2^ for 2-chamber to 3180 cm^2^ for 5-chamber, and Cell Factory 4-chamber has a surface area of 2528 cm^2^. The cell expansion ratio using multilayered flasks has been reported to be between 4.11-fold and 316.25-fold (Table 2). Four studies [22, 23, 26, 36] achieved an expansion ratio below 20-fold, and three studies [6, 17, 37] reported an expansion ratio above 100-fold using a multilayered flask (Figure 4).

### 3.5. Large-Scale Expansion Using Bioreactor

Many types of bioreactors, including hollow fiber bioreactor (Quantum Cell Expansion System) [19, 38], stirred tank bioreactor (UniVessel® SU bioreactor [28], Mobius® bioreactor [21], Celligen 310 bioreactor [26, 31, 35], Vertical Wheel bioreactor [27], Biostat Qplus bioreactor [27], and BioFlo 110 bioreactor [32]), and multiplate bioreactor (Pall Life Sciences Xpansion Multiplate Bioreactor) [39], have been tested for large-scale expansion of MSCs. Most studies used commercially available bioreactors with capacity ranging from 1.3 l to 50 l except Egger et al. who built their own stirred tank bioreactor for the expansion of AT-MSCs [20]. As the bioreactor capacity increased, the number of cells seeded and total cell yield also increased. Typically, microcarriers, including collagen-coated microcarriers, plastic P102L microcarrier, Cultispher S microcarrier, and Synthemax II microcarrier, were used to provide the culture surface for cells to attach and grow. The cell expansion ratio was reported to be between 1.85-fold and 42.67-fold depending on the bioreactor and culture protocol used.

### 3.6. Large-Scale Expansion Using Spinner Flask

Several types of spinner flasks, including the Bellco spinner flask [18, 24, 25, 30, 31, 33], Techne spinner flask [7, 26], Corning spinner flask [29], and Cellspin spinning bottle [16] with capacity ranging from 100 ml to 125 ml, have been used for large-scale expansion of MSCs. Microcarriers were used to provide the growth surface for cell proliferation. Different types of microcarriers, i.e., plastic P102L microcarrier, Cultispher S microcarrier, Cytodex 3 microcarrier, and Synthemax II microcarrier, were used in these studies. The cell expansion ratio has been reported to be between 2.60-fold and 21.00-fold.

### 3.7. Large-Scale Expansion Using Roller Bottle

A roller bottle is a cylindrical vessel that requires a roller track to gently rotate them. Only one study reported the use of roller bottles for MSC large-scale expansion. Tozetti et al. seeded 4.25 × 10^6^ MSCs in a 2125 cm^2^ roller bottle containing 200 ml of culture medium for 6 days to yield 2.98 × 10^7^ cells, achieving an expansion ratio of 7.01-fold [26].

## 4. Discussion

MSCs have great therapeutic potential and have been tested in many clinical trials. It is very important to produce MSCs in a large scale to meet clinical demands. One of the most crucial aspects to achieve this is the selection of a culture medium to support rapid MSC expansion without compromising its therapeutic potential. From the literature search, we found that FBS, HPL, and defined medium are frequently used for MSC expansion. FBS helps in cell adhesion by providing the cell attachment factors and is rich in growth factors that stimulate cell growth [40, 41]. However, there are concerns about its safety as MSCs cultured with FBS may trigger immunoreaction in recipients because of the transfer of animal protein and animal pathogen [14]. In addition, FBS has high batch-to-batch variation which leads to inconsistency in cell expansion [42]. These drawbacks indicated that the use of FBS should be avoided if possible. Human serum and defined medium are alternatives for FBS for large-scale expansion of MSCs. The main disadvantage of human serum and defined medium is the cost. In addition, human serum has batch-to-batch variation, and most of the defined medium require an extra culture surface coating step to improve cell attachment. Nonetheless, data extracted from the studies showed that MSCs were able to maintain its phenotype and trilineage differentiation potential as well as the immunomodulatory properties regardless of the culture medium, bioprocessing strategies, and serum supplement used, fulfilling the minimum criteria proposed by the ISCT [43].

Govindasamy et al. and Haack-Sørensen et al. compared the large-scale expansion of MSCs using FBS and HPL [17, 19]. Data from these studies showed that HPL significantly increased the cell yield and shortened the population doubling time compared to FBS without compromising the cell viability or altering their phenotype and trilineage differentiation potential. Similar results were reported in the study by Picken et al. that compared FBS with defined medium [18]. Melkoumian et al. compared 2 defined mediums, i.e., Mesencult™-XF medium and Corning® stemgro® hMSC medium [29]. The authors found that the Corning® stemgro® hMSC medium gave higher fold of cell expansion compared to Mesencult™-XF medium. None of the large-scale expansion studies compared HPL and defined medium. However, using a small-scale culture system, Riis et al. found that HPL gave the highest cell yield, followed by FBS, while the StemPro® MSC SFM XenoFree medium failed to maintain AT-MSC expansion beyond passage 5 [44]. Similarly, Oikonomopoulos et al. reported that expansion with HPL resulted in the highest cell proliferation, followed by StemPro® MSC SFM XenoFree medium and FBS [45]. Surprisingly, the authors observed that HPL failed to maintain BM-MSC and AT-MSC immunosuppressive properties. However, several previous studies reported contradicting results whereby Menard et al. found that BM-MSCs and AT-MSCs cultured with HPL were able to maintain their immunosuppressive properties compared to BM-MSCs cultured with FBS [46]. Tsai et al. reported that BM-MSCs cultured with HPL supplemented medium were able to maintain the immunosuppressive properties [35]. Thus, HPL is superior compared to FBS and defined medium for large-scale expansion of MSCs as it increases the proliferation of MSCs without compromising the characteristic and plasticity of the cells. Furthermore, the use of HPL also reduces the risk of animal pathogen transmission and animal protein transfer to host. Moreover, HPL is cheaper compared to defined medium that is still very costly right now. In the future, the cost of defined medium might reduce when the demand increases.

There were four bioprocessing strategies used to archive large-scale production of MSCs, i.e., multilayered flask, spinner flask, roller bottle, and bioreactor. Each bioprocessing strategy has its own advantages and disadvantages (Table 3). Generally, a bioreactor allows fully automated cell bioprocessing with higher efficiency. The multilayered flask, spinner flask, and rotating bottle are manual bioprocessing strategies with lower efficiency. Since the spinner flask, roller bottle, and multilayered flask require substantive manual manipulations, more manpower are needed when these culture systems are used. Among the four bioprocessing strategies, the multilayered flask is the only static cell culture system, while the rest are dynamic cell culture systems. A dynamic culture system creates shear stress to cells as it involves mechanical agitation of the culture medium or culture vessel to allow more efficient nutrient transfer. Regardless of the bioprocessing strategies, the cell culture vessels used come in multiple dimensions, from milliliters to liters. A smaller vessel is suitable for large-scale expansion of autologous MSCs to meet a relatively lower cell number requirement while a larger vessel is ideal for the expansion of allogenic MSCs to maximize the cell yield to produce thousands of therapeutic doses per batch production.

The bioreactor is very useful for ultra-large-scale MSC expansion as it allows more control over the culture environment such as oxygen concentration. The bioreactor is relatively difficult to operate but allows easier monitoring and scaling up using a single vessel of different capacities to generate the desired quantity of cells. Before large-scale expansion in a bioreactor, most studies expanded MSCs in standard culture flasks to obtain sufficient cells for seeding in the bioreactor. Nonetheless, two studies expanded MSCs starting from passage 0 in the bioreactor and reported a cell expansion ratio of 1.66-fold to 8.15-fold (AT-MSCs from seeded stromal vascular fraction (SVF)) and 4.11-fold (BM-MSCs from seeded bone marrow mononuclear cells (BMMCs)), respectively [19, 22]. Cunha et al. found that bioreactors can be used for large-scale expansion of AT-MSCs and BM-MSCs without compromising the cell viability, surface marker expression, and differentiation potential, even though the positive expression of CD105 dropped below 95% (88% for BM-MSCs and 92% for AT-MSCs) [28]. Similarly, several other studies also reported a reduction in the expression of CD90 and CD105 on MSCs expanded using bioreactors [26, 31, 32]. A few studies that used a spinner flask for MSC expansion also found that the expression of CD90 and CD105 decreased [31, 33]. The authors postulated that this is likely due to cell damage caused by shear stress or enzymatic cell detachment process. CD105^−^ MSCs have been reported to be more prone to differentiate into adipocytes and osteocytes and are more efficient in suppressing the proliferation of CD4^+^ T-cells compared to CD105^+^ MSCs [47]. A separate study found that CD105^−^ MSCs have poorer cardiac regeneration potential compared to CD105^+^ MSCs [48]. CD90^−^ MSCs have been linked with weaker immunosuppressive activity and enhanced osteogenic and adipogenic differentiation [49, 50]. Thus, the loss of CD90 and CD105 expression after bioreactor and spinner flask expansion might enhance the potency of the MSCs in treating certain diseases.

There are several important parameters to optimize when using bioreactors including oxygen concentration, frequency of medium change, and rotation speed of the impeller. It has been reported that the expression of MSC surface markers decreased due to the shear stress [26, 28, 31, 32]. Importantly, the cell loading and harvesting of specific bioreactors need to be improved as Haack-Sørensen et al. reported 30% cell loss during cell loading and another 30% during cell collection [19] and Luyten et al. found that the cell harvesting was as low as 45% [51]. The level of dissolved oxygen partial pressure in culture medium can affect the expansion of MSCs. Kwon et al. found that hypoxic culture enhanced MSC proliferation by increasing the number of cells in the S phase of the cell cycle [52]. HIF-1a is an important factor for cell adaptation to varying oxygen concentrations and usually highly expressed during hypoxia. HIF-1a has been linked with higher MSC proliferation and survival in hypoxic condition [53, 54]. Only one study compared the large-scale expansion of MSC in hypoxic and normoxic conditions. Egger et al. found that hypoxic culture increased the proliferation and enhanced the chondrogenic and adipogenic differentiation potential of MSCs but suppressed the osteogenic differentiation potential [20]. Similar studies have been conducted by Longaker et al. [34] and Dos Santos et al. [55] using small-scale cultures. Longaker et al. found that hypoxia condition diminished *in vitro* chondrogenesis and osteogenesis of AT-MSCs, while Dos Santos et al. did not find any difference in the BM-MSC osteogenic and adipogenic differentiation potential in hypoxic and normoxic cultures. Thus, even though hypoxic culture increases the proliferation of MSCs, the use of low oxygen concentration in culture must be carefully monitored as it might alter the cell therapeutic potential.

The spinner flask and roller bottle can be considered as a simpler and smaller scale bioreactor [14]. The spinner flask and roller bottle are less complicated and require more manual manipulation compared to the bioreactor. Just like the bioreactor, the rotation speed for the spinner flask impeller and roller bottle needs to be optimized to reduce shear stress that may damage the cells.

Generally, it appears that the bioreactor and multilayered flask are the most effective bioprocessing strategies as it has the potential to achieve an expansion ratio 20-fold and above. However, for the multilayered flask, the expansion ratio varies greatly from study to study whereby some of the studies reported an expansion ratio below 20-fold and a few studies achieved above 100-fold expansion ratio. The higher expansion ratio in these studies is likely due to the low initial seeding density [6, 17, 37]. For example, Nekanti et al. seeded 1.27 × 10^6^ cells (1000 cells/cm^2^) in a CellSTACK 2-chamber and yielded 2.48 × 10^8^ cells, achieving an expansion ratio of 195.28-fold [6]. In a different study, the authors seeded 5.09 × 10^6^ cells (4000 cells/cm^2^) in the same multilayered flask and yielded 3.64‐5.65 × 10^7^ cells to achieve 7.15-fold to 11.10-fold expansion ratio [23].

Most of the studies characterized the MSCs based on the ISCT guideline by checking at the phenotype and trilineage differentiation potential. However, this is not sufficient as the cell therapeutic potential, e.g., immunomodulatory property, is not reflected in these characterization techniques. Thus, many studies performed the immune-suppression assay to determine the functionality of expanded cells. Furthermore, some studies also performed extra experiments to detect the chromosome abnormality, genomic stability, and expression level of tumor markers to ensure the safety of the expanded cells. It is highly recommended to perform these extra testing, especially the potency assay, when the MSCs expanded in large scale are intended for clinical use.

## 5. Conclusion

Large-scale expansion of MSCs is commonly done using a multilayered flask, spinner flask, and bioreactor. Nonetheless, optimization of a few parameters, including cell seeding density, impeller agitation speed, oxygen partial pressure, medium formulation and feeding strategy, pH, and microcarrier selection, is crucial to ensure the development of a sustainable and reproducible platform to produce cells that suit clinical applications. In some instances, e.g., expansion of autologous cells that normally require a lower cell number, a multilayered flask is sufficient for upscaling in a cost-effective manner while a bioreactor is more suitable for ultra-large-scale expansion. However, none of the studies mentioned significant loss of cell characteristics and functionality when the bioreactor, spinner flask, roller bottle, and multilayered flask were used.

## Figures and Tables

**Table 1:** Summary of the articles describing the large-scale expansion of MSCs.

Reference	Bioprocessing method	Cell source	Cell culture medium	Initial cell seeding	Culture period (days)	Final cell yield	Expansion ratio	Doubling time (h)	MSC characterization	Other key findings
[6]	CellSTACK 2-chamber	WJ-MSCs	DMEM-KO with 10% FBS and 2 ng/ml bFGF	1.27*E* + 06	5	2.48*E* + 08	195.28	15.77	(i) Positive for CD44, CD73, CD90, CD105, CD146, and CD166 (ii) Negative for CD34, CD45, and HLA-DR (iii) Maintained the trilineage differentiation potential (iv) Maintained the capability to inhibit T-cell proliferation (v) No chromosome abnormality (vi) No hTERT expression (vii) No changes in expression of p53, p21, p16, and c-Myc	(i) Presence of bFGF boosted the cell growth

[17]	CellSTACK 5-chamber	DP-MSCs	DMEM-KO with 10% HPL	3.18*E* + 06	11	4.98*E* + 08	156.60	36.21	(i) >80% positivity for CD44, CD73, and CD90 (ii) <2% positivity for CD34, CD45, and HLA-DR (iii) Maintained the trilineage differentiation potential (iv) Maintained the expression of stem cell markers, i.e., SOX2, OCT4, and NANOG	(i) Supplementation with HPL gave higher cell yield
			DMEM-KO with 10% FBS			3.2*E* + 08	100.63	39.68		

[22]	Cell Factory 4-chamber	BM-MSCs	*α*MEM with 10% HPL and 2 IU/ml heparin	1.9*E* + 08 (BM-MNCs)	13.5	7.8*E* + 08	4.11	159.02	(i) >95% positivity for CD73, CD90, and CD105 (ii) <2% positivity for HLA-DR, CD14, CD19, CD34, and CD45 (iii) Maintained the trilineage differentiation potential	

[23]	CellSTACK 2-chamber	BM-MSCs	*α*MEM supplemented with 8% HPL and 1 IU/ml heparin	5.09*E* + 06	7	5.28*E* + 07	10.37	49.79	(i) >95% positivity for CD73, CD90, CD105, and HLA-ABC (ii) <1% positivity for CD3, CD34, and CD45 (iii) Maintained the trilineage differentiation potential	(i) Yield decreased for MSCs cultured with 10% HPL for 7 days likely due to cell detachment caused by culture over confluence
			*α*MEM supplemented with 8% HPL and 1 IU/ml heparin		5	4.69*E* + 07	9.22	37.44		
			*α*MEM supplemented with 10% HPL and 1 IU/ml heparin		7	3.64*E* + 07	7.15	59.21		
			*α*MEM supplemented with 10% HPL and 1 IU/ml heparin		5	5.65*E* + 07	11.10	34.56		

[36]	Hyperflask	VC-MSCs	DMEM/F-12 with 10% FBS and 3 ng/ml bFGF	5.16*E* + 06	4	7.4*E* + 07	14.26	23.75	(i) >95% positivity for CD73, CD90, and CD105 (ii) <1% positivity for CD34, CD45, CD79, and HLA-DR (iii) Maintained the trilineage differentiation potential (iv) Normal karyotype	

[37]	Cell Factory 4-chamber	BM-MSCs	*α*MEM with 10% FBS	0.8*E* + 06	15	2.5*E* + 08	316.25	49.80	(i) Maintained the trilineage differentiation potential	

[7]	Spinner flask+Cytodex 3 microcarrier	BM-MSCs	*α*MEM with 15% FBS	6.25*E* + 06	7	2.41*E* + 07	3.86	86.28	(i) >90% positivity for CD73, CD90, and CD105 (ii) Maintained the trilineage differentiation potential (iii) No chromosome abnormality (iv) Maintained the capability to inhibit lymphocyte proliferation	

[16]	Spinning bottle+CultiSpher-G microcarrier	WJ-MSCs	MEM/F12 with 10% FBS and 10 ng/ml bFGF	5*E* + 06	6	1.3*E* + 07	2.60	104.46	(i) >95% positivity for CD44, CD73, CD90, and CD105 (ii) Low expression of CD31 and CD45 (iii) No chromosomal abnormality (iv) Maintained the trilineage differentiation potential (v) High expression of embryonic markers (OCT4, SOX2, NANOG, and C-MYC)	(i) The cells expanded with spinner flask were more efficient in promoting *in vivo* wound healing compared to those expanded with culture flask

[18]	Spinner flask+plastic microcarrier	BM-MSCs	DMEM with 10% FBS	3*E* + 06	6	8.58*E* + 06	2.86	94.99	(i) >99% positivity for CD73, CD90, and CD105 (ii) <1% positivity for HLA-DR (iii) Maintained the trilineage differentiation potential	(i) Serum-free medium enhanced cell growth
	Spinner flask+plastic microcarrier coated with fibronectin		PRIME-XV™ SFM			3.01*E* + 07	10.03	43.29		

[24]	Spinner flask+Cultispher S microcarrier	PD-MSCs	DMEM-HG with 10% FBS	2.0*E* + 06	10	5.4*E* + 06	2.7	167.5	(i) Simultaneous expression of CD73, CD90, and CD105 in 89% of MSCs cultured with FBS and 86% in those cultured with HPL (ii) Low expression of negative markers for both mediums (iii) Sox9, ALP, BMP2, and WNT5A were upregulated in MSCs cultured with HPL compared to those cultured with FBS (iv) *In vivo* study showed that MSCs cultured with HPL formed more matured mineralization tissue compared to those cultured with FBS which form fibrous tissue	(i) Shear stress affected expression of percentage of positive surface marker (ii) MSCs cultured with HPL are more potent in bone formation *in vivo*
			DMEM-HG with 10% HPL	2.0*E* + 06	10	10.4*E* + 06	5.2	100.9		

[25]	Spinner flask+plastic microcarrier	WJ-MSCs	DMEM-LG with 5% UltraGRO™ and 2 IU/ml heparin	4*E* + 06	5.5	2.8*E* + 07	7.00	47.02	(i) >95% positivity for CD73, CD90, and CD105 (ii) Maintained the trilineage differentiation potential	

[29]	Spinner flask+Corning Synthemax II microcarrier	BM-MSCs	Mesencult™-XF	7.5*E* + 05	7	3.75*E* + 06	5.00	72.35	(i) >95% positivity for CD73 and CD105 (ii) <1% positivity for CD14 and CD45 (iii) Normal karyotype (iv) Maintained the trilineage differentiation potential	(i) Culture with Stemgro hMSC gave higher cell yield and expansion ratio and lower population doubling time
			Stemgro hMSC			5.25*E* + 06	7.00	59.84		

[30]	Spinner flask+microcarrier	BM-MSCs	StemPro MSC SFM Xenofree	4*E* + 06	8	2.88*E* + 07	7.20	67.42	(i) Cells expressed high level of CD105, CD73, and CD90 and lower level of CD31, CD80, and HLA-DR (ii) Maintained the trilineage differentiation potential	
		AT-MSCs		1.5*E* + 06		1.52*E* + 07	10.13	57.80		

[31]	Spinner flask+Cultispher® S microcarrier coated with CELLstart CTS solution	WJ-MSCs	StemPro MSC SFM XenoFree	4*E* + 06	5	1.92*E* + 07	4.80	53.03	(i) Maintain expression of CD90 and CD73 postexpansion (ii) Expression of CD105 decreased postexpansion, probably due to cell damage by shear stress (iii) Low expression of CD31, CD80, and HLA-DR (iv) Maintained the trilineage differentiation potential (v) Maintained the capability to inhibit lymphocyte proliferation	(i) Cells cultured with bioreactor have higher expansion ratio compared to those expanded using the spinner flask
	2.5 l Celligen 310 bioreactor+Cultispher® S microcarrier coated with CELLstart CTS solution			2*E* + 07	4	1.12*E* + 08	5.60	38.63		

[33]	Spinner flask+plastic microcarrier coated with CELLstart CTS solution	BM-MSCs	StemPro MSC SFM XenoFree	4*E* + 06	14	1.6*E* + 07	4.00	168.00	(i) >95% positivity for CD73 and CD105 (ii) <2% positivity for CD31, CD80, and HLA-DR (iii) 92% and 82% CD90 positivity for BM-MSCs and AT-MSCs, respectively, likely due to damage to the cells caused by longer enzymatic cell detachment process or shear stress (iv) Maintained the trilineage differentiation potential	
		AT-MSCs				1.12*E* + 07	2.80	226.20		

[56]	Spinner flask+Cytodex 3 microcarrier	F-MSCs	*α*MEM with 10% FBS	9.6*E* + 06	7	8.5*E* + 07	8.85	53.40	(i) >95% positivity for CD73, CD90, and CD105 (ii) <1% positivity for CD34	(i) Cells cultured with spinner flask have better osteogenic differentiation potential compared to those cultured in culture flask

[19]	Quantum Cell Expansion System	AT-MSCs	*α*MEM with 10% FBS	2.1*E* + 07	21	1.19*E* + 08	5.67	201.4	(i) >95% positivity for CD90, CD73, CD105, CD13, CD166, and CD29 (ii) <5% positivity for CD45, CD19, CD31, and HLA-DR (iii) No genomic instability (iv) Maintained the trilineage differentiation potential	(i) HPL is superior compared to FBS (ii) 1.66-fold to 8.15-fold of AT-MSCs was harvested from SVF seeded at P0
			*α*MEM with 5% heparin-free HPL		6	6.05*E* + 08	28.81	29.70		

[20]	Stirred tank reactor	AT-MSCs	*α*MEM with 10% HPL and 1 IU/ml heparin in 21% O_2_	1.3*E* + 07	6	2.4*E* + 07	1.85	162.80	(i) Positive for CD73, CD90, and CD105 (ii) Negative for CD14, CD20, CD35, CD45, and HLA-DR (iii) Maintained the trilineage differentiation potential	(i) Hypoxic cells displayed slightly poorer osteogenic differentiation potential and slightly better adipogenic and chondrogenic differentiation potential
			*α*MEM with 10% HPL and 1 IU/ml heparin in 5% O_2_			2.9*E* + 07	2.23	124.40		

[21]	Mobius® 50 l bioreactor+collagen-coated microcarrier	BM-MSCs	*α*MEM supplemented with 5% HPL and 2 IU/ml heparin	3*E* + 08	11	1.28*E* + 10	42.67	48.75	(i) >95% positivity for CD105, CD90, CD73, and CD44 (ii) <5% positivity for CD19, CD34, CD 11b, CD79a, CD45, and CD14 (iii) Low expression of HLA-DR (iv) Maintained the trilineage differentiation potential (v) Maintained the immunosuppressive properties	

[27]	Vertical Wheel bioreactor+Synthemax II microcarrier	BM-MSCs	MesenCult-XF with 0.025% (*v*/*v*) antifoam C emulsion	5.5*E* + 07	14	6.6*E* + 08	12.00	93.72	(i) Positive for CD44, CD73, CD90, CD105, and CD166 (ii) Negative for CD34 and CD45 (iii) Low expression of HLA-DR (iv) Maintained the trilineage differentiation potential	(i) Cells cultured with Vertical Wheel bioreactor have significantly lower expression of HLA-DR compared to those cultured with Biostat Qplus bioreactor
	Biostat Qplus bioreactor+Synthemax II microcarrier			6.25*E* + 06		6.88*E* + 07	11.00	97.10		

[28]	2 l UniVessel® SU bioreactor+Synthemax® II microcarrier	BM-MSCs	Mesencult™-XF	2.5*E* + 07	7	4.22*E* + 08	16.88	41.20	(i) >95% positivity for CD44, CD73, and CD90 (ii) <5% positivity for CD45, CD34, CD14, CD19, and CD11b (iii) Positivity of CD105 was 88% and 92%, respectively, for BM-MSCs and AT-MSCs (iv) Maintained the trilineage differentiation potential	
		AT-MSCs				5.06*E* + 08	20.24	38.72		

[32]	1.3 l BioFlo 110 bioreactor+plastic microcarrier coated with CELLstart CTS solution	BM-MSCs	StemPro MSC SFM XenoFree	5*E* + 05	7	1.1*E* + 08	22.00	21.59	(i) >90% positivity for CD73 (ii) Expression of CD90 and CD105 decreased to 74% and 39%, respectively, for BM-MSCs (iii) Expression of CD90 dropped to 64% (from graph) for AT-MSCs (iv) <2% positivity for CD31, CD80, and HLA-DR	
		AT-MSCs				4.5*E* + 07	9.00	25.88		

[35]	2.5 l Celligen 310 bioreactor+Fibra-Cel® disk	BM-MSCs	*α*MEM with 10% FBS	1.0*E* + 07	9	9.2*E* + 07	9.20	67.47	(i) >90% positivity for CD44, CD90, and CD105 (ii) Maintained the trilineage differentiation potential	

[38]	Quantum Cell Expansion System bioreactor	WJ-MSCs	F12K: DMEM-LG (1 : 1) with 10% FBS	2.1*E* + 07	7	4.0*E* + 08	19	39.5	(i) >99% positivity for CD44, CD73, CD90, and CD105 (ii) <1% positivity for CD45, CD34, CD11b, CD19, and HLA-DR (iii) MSCs maintained the trilineage differentiation potential (iv) Maintained the capability to inhibit lymphocyte proliferation (v) No alteration in karyotype	(i) There was no difference in cell proliferation and growth properties between MSCs cultured in flask and bioreactor

[39]	Pall Life Sciences Xpansion Multiplate Bioreactor	PD-MSCs	DMEM-HG with 10% FBS	1.6*E* + 08	7	5.35*E* + 08	3.34	96.47	(i) >90% positivity for CD73, CD90, and CD105 (ii) <5% positivity for CD45, CD20, CD14, and CD34 (iii) Maintained the trilineage differentiation potential	(i) 55% cell lost during the downstream process

[26]	HYPERFlasks®	WJ-MSCs	*α*MEM with 15% AB human serum	3.44*E* + 06	11	4.47*E* + 07	12.99	71.36	(i) Positive for CD73, CD90, and CD105 (ii) Negative for CD12, CD31, CD34, CD45, and HLA-DR (iii) Cells cultured with HYPERFlasks® showed reduction in CD73 expression (iv) Cells cultured with bioreactor showed reduction in CD105 expression, likely due to cell damage by shear stress (v) Maintained the trilineage differentiation potential (vi) Maintained the chromosome stability (vii) Maintained the immunosuppressive properties	
	Roller bottle			4.25*E* + 06	6	2.97*E* + 07	7.01	68.45		
	Spinner flask+plastic microcarrier			2*E* + 06	8	4.2*E* + 07	21.00	32.78		
	2.5 l Celligen 310 bioreactor+plastic microcarrier			8*E* + 06	7	7.92*E* + 07	9.90	50.79		

MSCs: mesenchymal stem cells; BM-MSCs: bone marrow-derived MSCs: BMNCs: bone marrow mononuclear cells; WJ-MSCs: Wharton's jelly-derived MSCs; AT-MSCs: adipose tissue-derived MSCs; PD-MSCs: periosteum-derived MSCs; VC-MSCs: villous chorion-derived MSCs; DP-MSCs: dental pulp-derived MSCs; F-MSCs: fetal MSCs; HPL: human platelet lysate: FBS: fetal bovine serum; bFGF: basic fibroblast growth factor.

**Table 2:** A summary of the expansion ratio achieved with different medium/serum supplement and bioprocessing strategies.

Medium/serum	Bioprocessing method (working volume)		Initial cell seeding	Final cell yield	Expansion ratio	Doubling time (h)	Reference
FBS	Multilayered flask	Hyperflask	5.16*E* + 06	7.36*E* + 07	14.26	23.75	[36]
		CellSTACK 5-chamber	3.18*E* + 06	3.20*E* + 08	100.63	39.68	[17]
		CellSTACK 2-chamber	1.27*E* + 06	2.48*E* + 08	195.28	15.77	[6]
		Cell Factory 4-chamber	8.00*E* + 05	2.53*E* + 08	316.25	49.80	[37]
	Spinner flask	Spinner flask (100 ml)	3.00*E* + 06	8.58*E* + 06	2.86	94.99	[18]
		Spinner flask (100 ml)	9.60*E* + 06	8.50*E* + 07	8.85	53.40	[56]
		Spinner flask (80 ml)	2.00*E* + 06	5.40*E* + 06	2.70	167.50	[24]
		Spinner flask (50 ml)	6.25*E* + 06	2.41*E* + 07	3.86	86.28	[7]
		Spinning bottle	5.00*E* + 06	1.30*E* + 07	2.60	104.46	[16]
	Bioreactor	Quantum Cell Expansion System	2.10*E* + 07	1.19*E* + 08	5.67	201.40	[19]
		Quantum Cell Expansion System	2.10*E* + 07	4.00*E* + 08	19.00	39.50	[38]
		Pall Life Sciences Xpansion Multiplate Bioreactor	1.60*E* + 08	5.35*E* + 08	3.34	96.47	[39]
		Fibrous bed bioreactor (1.75 l)	1.00*E* + 07	9.20*E* + 07	9.20	67.47	[35]

Human serum/human platelet lysate	Multilayered flask	Hyperflasks	3.44*E* + 06	4.47*E* + 07	12.99	71.36	[26]
		CellSTACK 2-chamber	5.09*E* + 06	5.28*E* + 07	10.37	49.79	[23]
		CellSTACK 2-chamber	5.09*E* + 06	4.69*E* + 07	9.22	37.44	[23]
		CellSTACK 2-chamber	5.09*E* + 06	3.64*E* + 07	7.15	59.21	[23]
		CellSTACK 2-chamber	5.09*E* + 06	5.65*E* + 07	11.10	34.56	[23]
		CellSTACK 5-chamber	3.18*E* + 06	4.98*E* + 08	156.60	36.21	[17]
		Cell Factory 4-chamber	1.90*E* + 08	7.80*E* + 08	4.11	159.02	[22]
	Spinner flask	Spinner flask (80 ml)	4.00*E* + 06	2.80*E* + 07	7.00	47.02	[25]
		Spinner flask (80 ml)	2.00*E* + 06	10.40*E* + 06	5.20	100.90	[24]
		Spinner flask (100 ml)	2.00*E* + 06	4.20*E* + 07	21.00	32.78	[26]
	Roller bottle	Roller bottle	4.25*E* + 06	2.98*E* + 07	7.01	68.45	[26]
	Bioreactor	Quantum Cell Expansion System	2.10*E* + 07	6.05*E* + 08	28.81	29.70	[19]
		Continuously stirred tank reactor (130 ml)	1.30*E* + 07	2.40*E* + 07	1.85	162.80	[20]
		Continuously stirred tank reactor (130 ml)	1.30*E* + 07	2.90*E* + 07	2.23	124.40	[20]
		Mobius® 50 l single-use bioreactor	3.00*E* + 08	1.28*E* + 10	42.67	48.75	[21]
		Stirred tank bioreactor (800 ml)	8.00*E* + 06	7.92*E* + 07	9.90	50.79	[26]

Defined medium	Spinner flask	Spinner flask (80 ml)	4.00*E* + 06	1.92*E* + 07	4.80	53.03	[31]
		Spinner flask (80 ml)	4.00*E* + 06	2.88*E* + 07	7.20	67.42	[30]
		Spinner flask (80 ml)	1.50*E* + 06	1.52*E* + 07	10.13	57.80	[30]
		Spinner flask (100 ml)	3.00*E* + 06	3.01*E* + 07	10.03	43.29	[18]
		Spinner flask (35 ml)	7.50*E* + 05	3.75*E* + 06	5.00	72.35	[29]
		Spinner flask (35 ml)	7.50*E* + 05	5.25*E* + 06	7.00	59.84	[29]
		Spinner flask (80 ml)	4.00*E* + 06	1.60*E* + 07	4.00	168.00	[33]
		Spinner flask (80 ml)	4.00*E* + 06	1.12*E* + 07	2.80	226.20	[33]
	Bioreactor	2 l Univessel® SU bioreactor (2 l)	2.50*E* + 07	4.22*E* + 08	16.88	41.20	[28]
		2 l Univessel® SU bioreactor (2 l)	2.50*E* + 07	5.06*E* + 08	20.24	38.72	[28]
		Stirred tank bioreactor (800 ml)	2.00*E* + 07	1.12*E* + 08	5.60	38.63	[31]
		Vertical Wheel bioreactor (2.2 l)	5.50*E* + 07	6.60*E* + 08	12.00	93.72	[27]
		Stirred tank bioreator (200 ml)	6.25*E* + 06	6.88*E* + 07	11.00	97.10	[27]
		1 l bioreactor (1 l)	5.00*E* + 06	1.10*E* + 08	22.00	21.59	[32]
		1 l bioreactor (1 l)	5.00*E* + 06	4.50*E* + 07	9.00	25.88	[32]

**Table 3:** Comparison between the large-scale bioprocessing strategies for mesenchymal stem cells.

Characteristic	Multilayered flask	Spinner flask	Rotating bottle	Bioreactor
Automation	No	No	No	Yes
Cost	Low	Medium	Medium	High
Technical difficulty	Low	Medium	Medium	High
Manpower needed	High	Medium	Medium	Low
Shear stress	No	Yes	Yes	Yes
Mass transfer	Low	High	High	High
Ease of scale-up	Low	High	Medium	High
Ease of monitoring	Low	Medium	Medium	High
Ease of cell collection	High	Medium to high	High	Medium to high
2D or 3D culture	2D	3D	2D	3D

## Additional Points

*Highlights*. (i) Mesenchymal stem cells required large-scale expansion for clinical use. (ii) Multiple bioprocessing strategies have been explored for large-scale expansion of mesenchymal stem cells (MSCs). (iii) Expanded cells maintain the MSC characteristics.
